# Grow against the flow: The impact of off-season vegetable production in Cambodia

**DOI:** 10.1371/journal.pone.0350969

**Published:** 2026-09-16

**Authors:** Liz Ignowski, Pepijn Schreinemachers, Mercy Mwambi, Uon Bonnarith, Ramasamy Srinivasan

**Affiliations:** 1 World Vegetable Center, Bangkok, Thailand; 2 World Vegetable Center, Arusha, Tanzania; 3 World Vegetable Center, Siem Reap, Cambodia; 4 World Vegetable Center, Tainan, Taiwan; Ardakan University, IRAN, ISLAMIC REPUBLIC OF

## Abstract

Vegetable production is strongly seasonal, creating income volatility for smallholder farmers and occasionally high prices for consumers. The ability to produce vegetables outside their regular growing season can enhance farmers’ incomes and income stability, yet empirical evidence remains limited. This study evaluates the impact of training farmers in Cambodia on off-season production technologies for yardlong beans, tomatoes, and brassicas during the hot-dry and wet seasons. Using survey data collected from 172 intervention and 179 control farmers two and three years after the training began, average treatment effects were estimated by using propensity score matching and inverse probability weighting. Trained farmers adopted an average of 3–4 more off-season technologies than the control group (p < 0.01). Two years after the intervention began, the share of trained farmers producing yardlong beans increased by 12 percentage points in the hot-dry season (with average production gains exceeding 100 kg) and by 46 percentage points in the wet season (with an average increase of about 700 kg). Corresponding revenue gains were approximately USD 50 and USD 300 per farmer, respectively (p < 0.01). Three years after the intervention began, revenue gains were lower, but still significant. Positive effects on tomato and brassica production and income were observed in the wet season, but not in the hot-dry season. These findings underscore the potential of off-season vegetable production to enhance rural livelihoods and improve year-round vegetable availability. The results provide evidence for stakeholders in Cambodia and comparable lower-income countries in Southeast Asia seeking to scale up off-season vegetable programs.

## Introduction

Vegetable production in Southeast Asia and elsewhere is strongly seasonal. Production is high, and prices are low during the primary production season, but production is low, and prices are high outside this season. The strong fluctuations in market supplies and prices create uncertainty for smallholder vegetable farmers and makes vegetables seasonally unaffordable for low-income consumers. Production technologies are available that enable more continuous production of vegetables year-round, thereby increasing vegetable availability [1,2]. These include hydroponic systems, which enable cultivation independent of soil quality and can extend production beyond the regular growing season [3]. Off-season vegetable production refers to growing vegetables under adverse climatic or economic conditions [4,5]. It may include growing vegetables under plastic cover, for which low-cost supplemental heating infrastructure has been shown to improve climate control in protected structures [6], and using heat-tolerant varieties. It may also include raised planting beds, seedling nurseries, and integrated pest management (IPM) targeting pests outside the regular growing season [7].

Adopting off-season practices can potentially improve rural livelihoods in several ways [8–10]. First, the supply of technologies and training is expected to increase farm productivity. Doing this in the off-season, when prices are high and supply is low, is expected to generate higher farm income and profits than producing in-season [5]. This is a crucial step towards rural and agricultural development, especially in Southeast Asia, where farming is a primary source of food and income for rural households [11].

Only a few studies have empirically tested whether adopting off-season technologies can generate additional income. Applying propensity score matching on cross-sectional data for 245 vegetable farmers in Bangladesh, a study showed that farmers trained in off-season vegetable production increased their income by 48% during the off-season after two years [5]. Also in Bangladesh, it was found that knowledge of good agricultural practices (GAP) (which often include new technologies), along with familiarity and training in vegetable production, significantly influenced growers’ interest in adopting GAP for vegetables [12]. A study in Côte d’Ivoire showed that increased adoption of agroecological practices benefited vegetable farmers in dealing with seasonality and decreased the adverse environmental effects of chemical use [13]. Furthermore, field experiments in India and Taiwan showed that technologies such as protected cultivation and drip irrigation increased crop yield, enhanced crop nutrient content, and improved farmers’ vegetable consumption [14,15]. A cross-sectional study in Nepal found that continued training, material support, and market information are key to sustaining the adoption of off-season production technologies [10].

This study aims to quantify the impact of training Cambodian farmers in off-season vegetable production using data from a project that trained 14,844 farmers. Cambodia presents an interesting case because it produces vegetables for a relatively short period of the year while importing many vegetables from neighboring countries during the monsoon and hot seasons, when local production is challenging because of flooding and heat. A previous study found a low uptake of new technologies by Cambodian rice farmers, but did not study off-season vegetable technologies [16]. Another study in Cambodia showed that enhancing crop production practices and improving the efficiency of resources can serve as a foundation for the sustainable intensification of agricultural production [17].

In this study, farmers were invited by the lead farmers in their village to attend training sessions on off-season vegetable production. The training included technologies such as improved varieties, plastic mulching, and raised beds to grow yardlong beans, brassicas, and tomatoes. Sessions were held throughout the year, and technical staff visited the farmers regularly. The adoption of off-season vegetable technologies is an understudied area that could greatly benefit smallholder farmers in many countries.

Applying propensity score matching and inverse probability weighting to correct for selection bias, this study finds that training vegetable farmers in off-season production creates positive impacts. Trained farmers were more likely to grow yardlong beans in the wet and hot-dry seasons, harvest more, and earn more. Farmers growing tomatoes and brassicas also experienced a positive impact on crop harvest and revenue in the wet season, but not in the hot-dry season. While the training focused on three specific vegetables, the skills could be applied to other crops. Lastly, the intervention increased the total revenue from all vegetables.

## Methods and data

### Intervention

The “Grow Against the Flow " project, funded by the Federal Ministry for Economic Cooperation and Development of Germany, trained Cambodian farmers in off-season, open field, vegetable production from July 2021 to June 2023. There was also one refresher training for the intervention group from July 2023 to May 2024. The project targeted high-value vegetables, including tomatoes, brassicas, and yardlong beans. Participating farmers were trained by the World Vegetable Center (WorldVeg), East-West Seed International (EWS), International Development Enterprises (iDE), and the General Directorate of Agriculture (GDA).

Cambodia has three distinct vegetable production seasons: (i) the cool-dry vegetable growing season from November to February, which is the primary season to produce vegetables on rainfed plots; (ii) the hot-dry season from March to May, when it is difficult to grow vegetables; and (iii) the wet season from June to October, when vegetable production is constrained by wetness and flooding. The hot-dry and wet seasons are the “off-seasons” targeted by the intervention.

The intervention utilized a farmer-to-farmer trainer approach to scale innovations. First, the project staff organized farmers into groups of 10–20 people, usually from the same village. Second, the groups and staff identified 3–5 lead farmers. Field demonstrations were established on the farms of these lead farmers. Criteria for selecting lead farmers included membership in farmer groups, personality traits (e.g., risk taker, cooperative), social position (e.g., a leader), and assets (e.g., owning at least 500 square meters of land). Women were encouraged to take the lead farmer role. Third, groups were invited to the demonstration site for training provided by technical officers. Field days were also organized at the demonstration sites. During these field days, group members and other people interested received training from the lead farmer with support from the technical officers. Each group received at least five training sessions on (i) land preparation, seedling production, and compost making (organic residues have been shown to be an effective, low-cost alternative potting medium for seedling and vegetable production [18]); (ii) transplanting, water management, and fertilizer management; (iii) pruning and weeding; (iv) insect pest and disease management based on IPM; and (v) record keeping from July 2021 to June 2023. They also had one additional refresher session on IPM and water and nutrient management between July 2023 to May 2024.

The demonstrated technologies included improved cultivars, grafted tomato seedlings, GAP, IPM, protective structures, and water conservation methods (drip irrigation). IPM practices included colored sticky traps, pheromones, and biopesticides. Protective structures included rain shelters, polyhouses, and low-cost plastic tunnels. The project demonstrated water conservation technologies for the hot-dry season, such as drip irrigation and mulching. Intervention farmers received seeds of commercially available heat-tolerant varieties of tomato (5 grams), various brassicas including pak choy and choy sum, which had a short duration maturing 25–35 days after sowing (10 grams), and yardlong bean (150–200 grams), accessories for drip irrigation systems, biopesticides (300–500gs of *Bacillus thuringiensis* (*Bt*), 300–500 ml of neem oil, 200-300g of Trichoderma), pheromone lures, sticky traps (5 blue and 5 yellow), plastic mulch (1 roll, 450 meters), trellising nets (3–5 rolls) and training materials (booklets, record-keeping book, leaflets). Materials were provided once at the beginning of the intervention, but seeds were provided three times at the start of each season.

### Sampling and data collection

**Fig 1:**
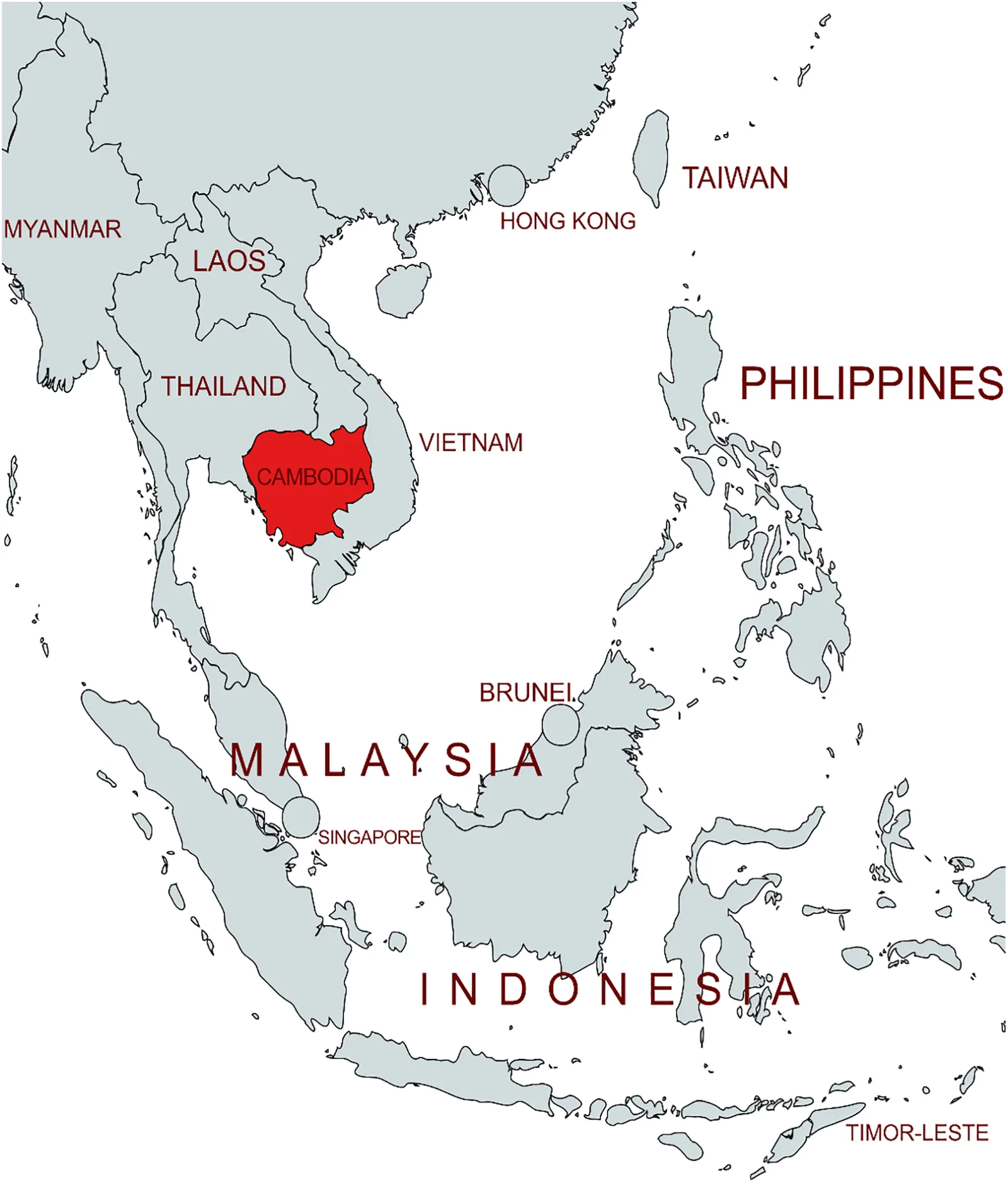
Location of the study sites showing Cambodia within Southeast Asia.

**Fig 2:**
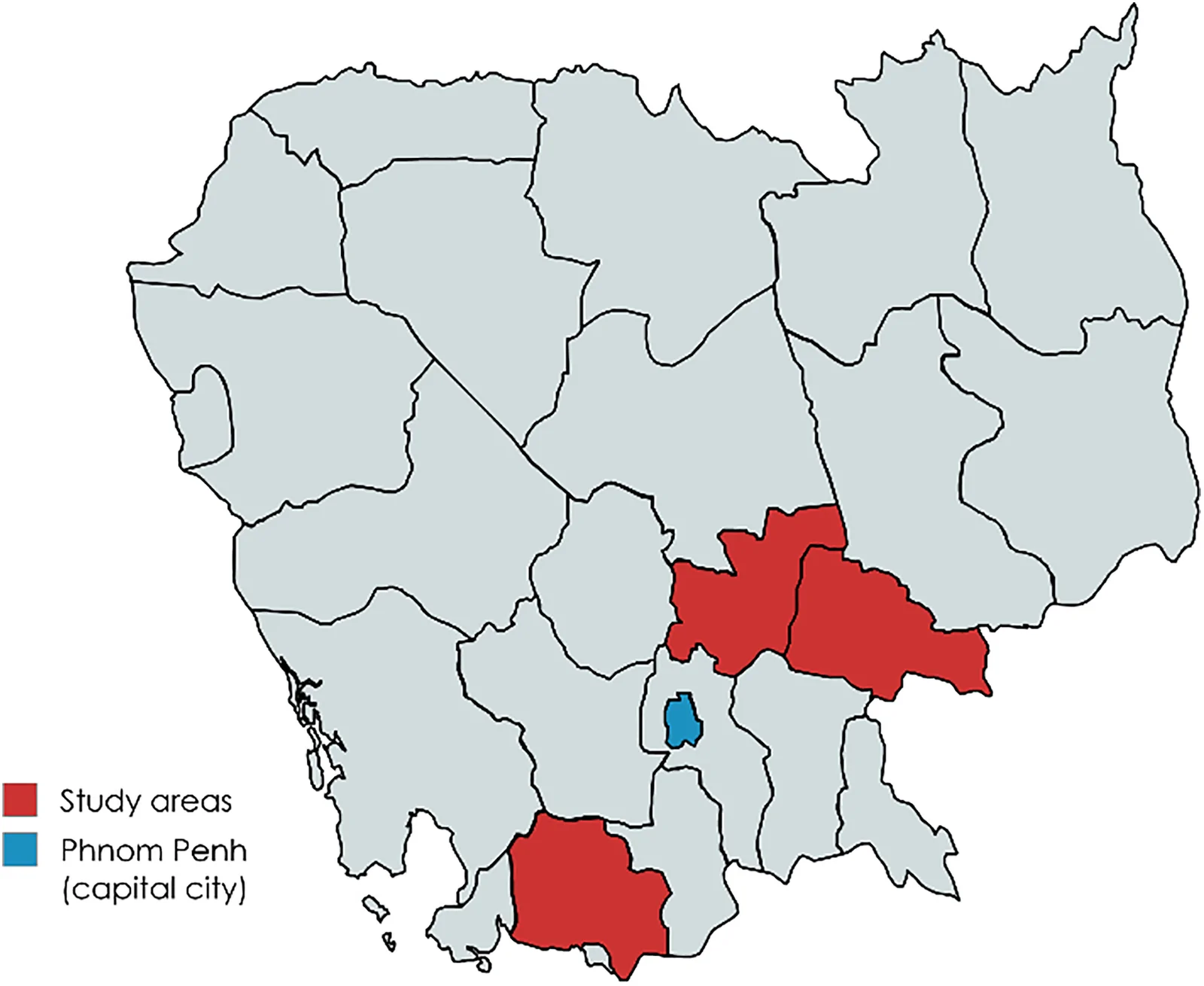
Location of the study sites showing the provinces of Kampong Cham, Kampot, and Tboung Khmum.

Data was collected in Kampong Cham, Kampot, and Tboung Khmum provinces of Cambodia (Figs 1 and 2). The sampling followed these steps: i) provinces and districts with vegetable production were identified with the help of the local partner; ii) provinces and districts had to be located in areas where the partners implemented the project; iii) intervention and control villages were identified by partners and had to be at least 5–10 km apart to minimize spillover effects; iv) village elders provided a list of vegetable farmers to participate in the survey from which the enumerators drew a random sample of 10–15 farmers.

Power calculations were done for a typical quasi-experimental design following Gertler et al. (2016) [19]. As the number of clusters (i.e., villages) and cluster sizes were fixed, power calculations were done by estimating the Minimum Detectable Effect (MDE) to measure a power of 80%. This suggested a target sample of 448 farmers. While a sample of 461 farmers was reached in the baseline, the sample size dropped to 387 in the first endline and 351 in the second endline, implying an attrition rate of 16% and 23%. The analytical sample had 351 farm household observations.

The study was reviewed and approved by the World Vegetable Center’s Institutional Biosafety and Research Ethics Committee (IBREC Reg. No. 2021−010) before the start of the data collection. Baseline data were collected in June 2021 before the intervention began. Informed consent was obtained verbally by the enumerators and recorded on tablet computers used to collect all survey data.

Baseline data collection relied on phone interviews for the because of the COVID-19 pandemic. The phone interviews were short, and asking detailed questions about production or revenues proved impossible. As a result, the baseline data had only limited value and were not used in the analysis. Endline data were collected through in-person interviews in June 2023 and again in June 2024 to test the sustainability of the intervention. Only these two endline surveys were used for the analysis.

Attrition bias was tested between the farmers surveyed in both endlines and those who were only surveyed in the 2023 endline based on the characteristics of age, gender, farm size, household head education, and province. A t-test was used to compare these variables. Only one variable was statistically different at the 5% level: farmers in the intervention group who dropped out were 58% female, while those who did not drop out were 34% female (p-value = 0.043). Attrition rates were not different between intervention and control groups, and in regression analysis, intervention assignment did not predict attrition. Hence, no evidence of attrition bias was found in the sample.

Data was collected using a structured questionnaire programmed on tablet computers using KOBO software. The questionnaire included questions on the above outcomes. Covariates included farm and household characteristics, training attended, crop production, and the use of technologies. Data were collected by a team of enumerators trained for a week to understand the survey and become familiar with the tablets. During these training sessions, the questionnaire was pre-tested and revised.

### Outcomes

The impact of the intervention was assessed on the following outcomes:

a. **Off-season technologies adopted:** the number of technologies (out of ten that were part of the training) that farmers adopted.
b. **Off-season vegetable production:** whether farmers produced tomatoes, yardlong beans, and brassicas during the off-seasons and the harvested quantity in kilograms (kg).
c. **Revenue from vegetable production:** the sales revenue from tomatoes, yardlong beans, and brassicas sold each season and the revenue from all vegetables combined.

### Propensity score matching

The study was designed as a quasi-experiment in which the intervention was not randomly assigned to groups of eligible households. The intervention’s impact was quantified using propensity score matching (PSM) and inverse probability weighting (IPW). These non-parametric methods do not assume that outcome variables fit a particular distribution. The analysis starts by regressing intervention assignment (control vs. intervention) on a set of independent characteristics that concurrently influence intervention assignment and outcomes. Covariates included the respondents’ age, sex, farm size, the education of the household head, and province.

PSM ranks households according to their conditional probability of being assigned a treatment [20]. The nearest neighbor method is used as the matching algorithm. The method identifies the most similar non-treated farmer for each treated farmer, and the most similar treated farmer for each non-treated farmer. Outcomes are then compared among matched pairs of treated and non-treated farmers based on their propensity scores. These differences are averaged over the entire sample to find the average treatment effect.

Inverse probability weighting was also used, which weights each individual farmer by the inverse probability of receiving actual treatment and does not match treated with untreated observations [21,22]. Farmers with a low predicted probability of receiving the intervention have a lower weight, while those with a high predicted probability have a higher weight. A farmer with a low predicted probability of being in the intervention but who was included will represent a larger group of farmers who did not receive the intervention and thus get a higher weight in calculating the average. The average treatment effect is then calculated as the difference between the weighted averages of intervention and control farmers.

### Testing of assumptions

When using propensity scores for matching or weighting, the distribution of covariates for the intervention and control groups must be balanced. Previous literature suggests three balancing tests [23]. First, an unpaired t-test was used to test that there were no significant differences in the average values of the covariates between the intervention and control groups after matching. Second, the pseudo-R^2^ was compared before and after matching, and should be low after matching. Lastly, the standardized percentage bias should be less than 20% for each covariate and less than 10% on average overall [24].

Another key requirement in using propensity scores is the common support condition, which ensures sufficient overlap in propensity scores between the intervention and control groups. This guarantees that for each trained farmer, there is at least one non-trained farmer with similar characteristics. To verify this, the propensity score distributions of both groups were visually assessed using plots.

Propensity score estimators assume that intervention assignment is based solely on observed covariates, such as age, farm size, education level, and province. However, hidden bias can arise if unobserved factors influence both program participation and the outcome variable [25]. Potential unobserved covariates include risk aversion, soil quality, and the ability to implement learned technologies. Some researchers suggest that if observed and unobserved covariates are correlated, the impact of unobserved factors may be mitigated. However, this assumption cannot be directly tested with the data. To address this concern, the Rosenbaum bounds test was used to assess the sensitivity of the results to hidden bias.

## Results

### Sample characteristics

Our sample of farmers had a mean age of around 50 years, with the average farmer in the intervention group being slightly younger than the control group (Table 1). The share of women in the control group (60%) was higher than in the intervention group (35%; p < 0.001). Both groups had a similar mean farm size of around 1.5 hectares (ha), and about 15% of farmers migrated seasonally. The results from Table 1 are based on the first endline data collection, not the baseline data collection (as explained above). The intervention group was more likely to grow vegetables in any of the three seasons than the control group, and the same goes for the crops of interest.

**Table 1:** Differences in the means of key characteristics of vegetable farmers, first endline (2023).

Characteristic	Pooled (n = 351)	Control (n = 179)	Intervention (n = 172)	p-value
Age (years)	50.15	51.81	48.42	0.007
Women farmers (proportion)	0.47	0.60	0.34	<0.001
Farmland (ha)	1.45	1.37	1.52	0.331
Province:				0.602
• Kampong Cham	0.33	0.33	0.35	
• Kampot	0.33	0.33	0.34	
• Tboung Khmum	0.34	0.34	0.31	
Grows vegetables (proportion):				
• June to October (wet season)	0.83	0.75	0.92	<0.001
• November to February (regular season)	0.72	0.58	0.87	<0.001
• March to May (hot-dry season)	0.31	0.22	0.40	<0.001
Grows target crops (proportion):				
• Yardlong bean	0.43	0.18	0.68	<0.001
• Brassica	0.23	0.16	0.31	<0.001
• Tomato	0.04	0.01	0.07	0.003
Off-season technologies used (#):				
• Overall	3.73	1.59	5.96	<0.001
• Wet season	3.48	1.43	5.62	<0.001
• Hot-dry season	2.84	0.96	4.80	<0.001

Almost all (99%) of the intervention group received training on yardlong bean production (Table 2). For brassica and tomato production, 96% of the intervention group participated in training on each crop. The respondents were also asked about the topics on which they had received training.

**Table 2:** Training participation of the intervention group as measured in both endlines, in the proportion of trained farmers (n = 172).

Training aspect	2023	2024
Received training on yardlong bean	0.99	0.99
Received training on brassicas	0.96	0.96
Received training on tomatoes	0.96	0.98
Topics trained:		
• Soil fertility	0.93	0.89
• Grafting	0.71	0.88
• Seeds and seed saving	0.84	0.91
• Pest and disease management	0.89	0.88
• Water management and irrigation	0.90	0.85
• Nutrition and healthy eating	0.83	0.79
• Postharvest management	0.82	0.74
• Marketing	0.90	0.87
• Record keeping	0.89	0.85
• Home gardening	0.82	0.67
• Land preparation	0.95	0.85

Land preparation and soil fertility were reported the most (95% and 93%), while grafting was reported the least (71%). The intervention group was also asked about their satisfaction with each training topic. Over 95% of respondents were more than satisfied with the training.

**Fig 3:**
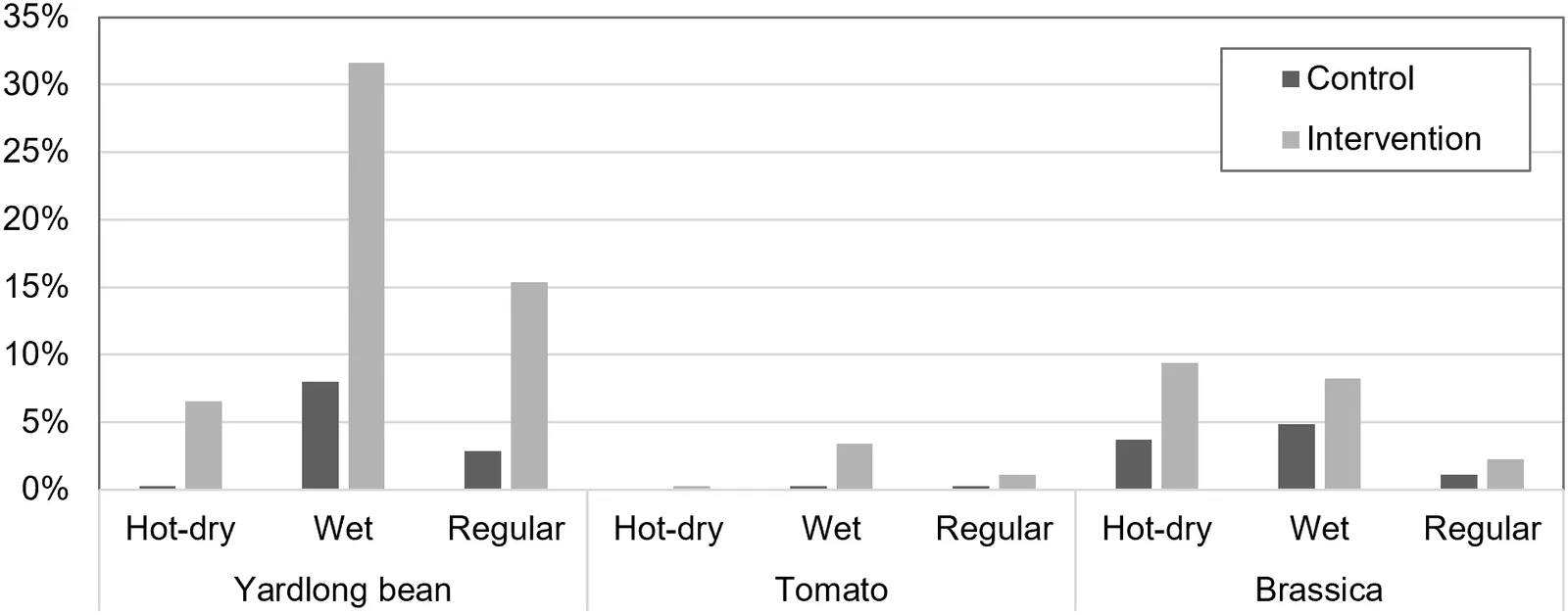
Share of control and intervention farmers producing target vegetables by season, first endline (2023; n = 351). Notes: Chinese cabbage, choysum, and pak choy are the three most common brassicas grown, but others asked about in the survey include Chinese mustard, headed cabbage, broccoli, cauliflower, radish, and kale.

The share of farmers growing crops in the twelve months before data collection in 2023 varied by crop and season (Fig 3). Yardlong beans were the most grown, followed by brassicas, then tomatoes. A larger share of farmers in the intervention group grew more of each crop in every season compared to the control group.

The intervention group increased its production area for yardlong beans during each of the three seasons from July 2021 to June 2023 (Table 3). For tomatoes, the production area was larger during the wet season (p = 0.01) and regular season (p < 0.10). The production area of brassica was only higher during the wet season (p < 0.10).

**Table 3:** Test of difference in means for area under target vegetables, m^2^.

Season/ year	Yardlong beans			Tomatoes			Brassicas		
	Control	Intervention	p-value	Control	Intervention	p-value	Control	Intervention	p-value
Hot-dry:									
2023	23.4	370.1	<0.001***	0.0	11.6	0.308	124.4	236.6	0.161
2024	24.2	399.5	<0.001***	0.0	2.9	0.308	53.4	157.5	0.024**
Wet:									
2023	158.9	1,019.4	<0.001***	5.6	78.8	0.011**	111.2	321.7	0.056*
2024	120.4	620.3	<0.001***	0.0	27.3	0.011**	142.2	137.9	0.937
Regular:									
2023	75.5	543.0	<0.001***	1.7	46.5	0.097	43.0	45.9	0.924
2024	30.5	409.8	<0.001***	0.0	17.4	0.052*	31.6	96.2	0.106

Notes: Control n = 180 and Intervention n = 172. Chinese cabbage, choysum, and pak choy are the three most common brassicas grown, but other brassicas include Chinese mustard, headed cabbage, broccoli, cauliflower, radish, and kale. *p < 0.10, ** p < 0.05, *** p < 0.01. p-value based on a t-test of means between control and intervention.

### Average treatment effects

The balancing requirement was met across all three tests (S1 Table in Supporting Information). The unpaired t-test revealed no significant differences in covariate means between the intervention and control groups. The pseudo-R² values decreased from 0.073 in 2023 and 0.065 in 2024 before matching to 0.006 and 0.009 after matching. Additionally, the standardized percentage bias for each covariate remained below 20%, while the mean absolute standardized bias was under 10%. These results indicate that propensity score estimators effectively reduced bias in observable characteristics between the intervention and control households. The results were mildly sensitive to hidden bias (S2 Table in Supporting Information). All lower bounds for both years had significance levels of p < 0.01 (except for brassica output in the wet season of 2023). The upper bounds became insignificant in 2023 (p > 0.10) if the gamma was increased by a factor of 1.8 for annual vegetable income. The upper bounds only became significant (p < 0.10) for brassica production in the wet season if the gamma was increased by a factor of 2.2. In 2024, the upper bounds became insignificant if the gamma was increased by a factor of 2.6 for yardlong bean production in the wet season, 2.2 for brassica production in the wet season, 1.4 for annual vegetable income. This does not indicate that the assumptions are violated. However, the propensity score distributions suggest a potential lack of overlap in 2023 (S1 Fig in Supporting Information; 2024 propensity score distributions are presented in S2 Fig in Supporting Information). To test the common support condition, observations whose propensity scores fell outside the region of overlap between the two groups were excluded from estimation. This included 12 control households in 2023 and 16 control households in 2024. These exclusions are a subset of control farmers who had characteristics sufficiently different from the intervention group. Keeping these observations could bias the average treatment effect estimate upward if they disproportionately represent farmers least likely to benefit from the intervention. Therefore, whether the average treatment effects are sensitive to excluding observations outside the common support region is assessed (S3 Table in Supporting Information). The coefficient of variation was used as a measure of sensitivity. The results show that technologies adopted, yardlong bean and tomato production in the wet season, and annual vegetable income were not very sensitive to alternative matching methods in 2023, with ATEs varying less than 3% around the mean. Brassica production was more sensitive, but its ATEs were generally not significant in 2023. In 2024, technologies adopted and yardlong bean production remained insensitive to alternative matching methods, with their ATEs varying less than 4% around the mean. Brassica and tomato production in the wet season and annual vegetable income were more sensitive, with an average variation of 10.4–12.2% around the mean ATE.

The first outcome of interest was the adoption of off-season technologies demonstrated to the intervention group. The average farmer in the intervention group adopted four more technologies (p < 0.01) than the average control farmer in the first endline (which was two years after the start of the intervention) (Table 4). This effect was maintained in 2024, although at a slightly lower level. Treatment effects for the adoption of each of the ten technologies were positive in each year. The use of improved varieties, water management, and foliar fertilizers was slightly lower in 2024 than in 2023 but was still significantly higher for the intervention than the control group. PSM and IPW gave similar results.

**Table 4:** Impact on the proportion of farmers using off-season technologies.

	2023		2024	
	PSM	IPW	PSM	IPW
IPM practices adopted (0-10)	4.32*** (0.25)	4.33*** (0.21)	3.34*** (0.25)	3.29*** (0.23)
Technologies adopted:				
• Pheromone traps	0.39*** (0.05)	-	0.33*** (0.05)	0.32*** (0.04)
• Barrier crops	0.50*** (0.04)	0.49*** (0.04)	0.46*** (0.05)	0.45*** (0.04)
• Sticky traps	0.29*** (0.04)	-	0.34*** (0.05)	0.32*** (0.04)
• Commercial biopesticides	0.51*** (0.05)	0.52*** (0.05)	0.52*** (0.06)	0.47*** (0.04)
• Improved varieties	0.69*** (0.05)	0.68*** (0.04)	0.38*** (0.06)	0.42*** (0.05)
• Water conservation	0.51*** (0.06)	0.48*** (0.05)	0.21*** (0.05)	0.21*** (0.04)
• Straw/plastic mulch	0.51*** (0.06)	0.49*** (0.05)	0.41*** (0.05)	0.43*** (0.04)
• Plastic tunnel	0.05* (0.03)	0.07** (0.03)	0.03 (0.02)	0.04* (0.02)
• Composted manure	0.31*** (0.06)	0.32*** (0.05)	0.32*** (0.06)	0.30*** (0.05)
• Foliar fertilizers	0.50*** (0.06)	0.53*** (0.05)	0.34*** (0.06)	0.34*** (0.05)

Notes: *p < 0.10, ** p < 0.05, *** p < 0.01. Coefficients for average treatment effects (ATE) are presented with standard errors in parentheses. n = 351 farmers for each year. Each cell in the PSM and IPW columns is a separate regression. Potential outcome (PO) mean is the average outcome that would have been observed in the treatment and control groups under certain conditions, regardless of whether the groups were treated or not.

The intervention had a positive impact on the share of farmers producing yardlong beans in each of the three seasons, from an increase of 12 percentage points in the hot-dry season to 46 percentage points in the wet season, all with p < 0.01 (Table 5). The intervention farmers were also more likely to grow tomatoes in the wet season (an increase of 6 percentage points in 2023 with p < 0.01). Once again, PSM and IPW gave similar results. The IPW model could not be estimated for all tomato variables because the variance was too low, as few farmers produced tomatoes.

**Table 5:** Impact on the proportion of farmers growing target vegetables by season.

Season/ estimation method	Yardlong beans		Tomatoes		Brassicas	
	2023	2024	2023	2024	2023	2024
Hot-dry:						
*•* PSM	0.12*** (0.03)	0.10*** (0.03)	0.01 (0.01)	0.01 (0.01)	0.03 (0.03)	-0.01 (0.02)
*•* IPW	0.12*** (0.02)	0.10*** (0.03)	0.01 (0.01)	-	0.03 (0.02)	-0.006 (0.02)
Wet:						
*•* PSM	0.46*** (0.06)	0.31*** (0.06)	0.06*** (0.03)	0.04** (0.02)	0.04 (0.05)	0.12*** (0.04)
*•* IPW	0.50*** (0.05)	0.32*** (0.05)	0.07*** (0.02)	-	0.09** (0.04)	0.10*** (0.04)
Regular:						
*•* PSM	0.23*** (0.05)	0.27*** (0.04)	0.01 (0.02)	0.03 (0.01)	0.06 (0.04)	-0.02 (0.04)
*•* IPW	0.26*** (0.04)	0.26*** (0.04)	0.02 (0.02)	-	0.09** (0.04)	-0.02 (0.04)

Notes: *p < 0.10, ** p < 0.05, *** p < 0.01. Coefficients for average treatment effects (ATE) are presented with standard errors in parentheses. Brassicas include Chinese cabbage, choysum, pak choy, Chinese mustard, headed cabbage, broccoli, cauliflower, radish, and kale. n = 351 farmers for each year.

Average treatment effects on vegetable output are positive for the quantity of yardlong beans produced in all seasons, ranging from 112 kg in the 2023 hot-dry season (p < 0.001) to 684 kg in the 2023 wet season (p < 0.001) (Table 6). This effect was also apparent in 2024 with the quantity of yardlong beans produced ranging from 51 kg in the 2024 hot-dry season (p < 0.001) to 310 kg in the 2024 wet season (p < 0.001) There are also positive treatment effects for the quantity of tomatoes produced in the wet season in 2024 (19 kg, p = 0.049). Also, during the wet season, there is a positive and significant impact on the amount of brassica produced, but only in 2024. The PSM and IPW results are similar.

**Table 6:** Impact on the output of target vegetables by season, in kilograms per farmer.

Season/ estimation method	Yardlong beans		Tomatoes		Brassicas	
	2023	2024	2023	2024	2023	2024
Hot-dry:						
• PSM	112.96***(29.65)	51.34***(17.95)	3.99(3.40)	1.71(1.70)	14.22(14.85)	−11.32(11.78)
• IPW	114.90***(30.022)	50.320***(16.32)	–	–	12.92(10.31)	−14.82(12.22)
Wet:						
• PSM	684.30***(98.02)	310.22***(57.75)	36.47(22.45)	19.79**(8.82)	−22.73(87.23)	107.58***(39.03)
• IPW	716.90***(92.39)	343.38***(61.76)	46.17**(23.450)	–	30.98(55.38)	102.42**(40.63)
Regular:						
• PSM	255.58***(69.09)	165.06**(74.13)	−4.42(37.78)	8.72*(4.89)	8.36(53.00)	32.23(58.93)
• IPW	300.353***(59.96)	183.52**(85.95)	6.89(25.68)	–	44.77(42.00)	37.18(59.99)

Notes: *p < 0.1, ** p < 0.05, *** p < 0.01. Coefficients for average treatment effects (ATE) are presented with standard errors in parentheses. The brassica group includes Chinese cabbage, choysum, pak choy, Chinese mustard, headed cabbage, broccoli, cauliflower, radish, and kale. n = 351 farmers per year.

The impact on crop revenues (Table 7) is similar to that on quantity produced. There was a positive and statistically significant effect on the revenue from yardlong beans in all seasons in 2023, ranging from around 50 USD per household in the hot-dry season to 300 USD in the wet season (all p < 0.01). There was a treatment effect of 18 USD per household (p < 0.10) for the total kilograms of tomatoes produced in the wet season. The IPW model did not converge for some outcomes. Also, during the wet season, there was a positive and significant impact on the revenue from brassicas of 50 USD per household (p < 0.01), but only in 2024. The total revenue from the three target crops had a positive and significant impact across all seasons, ranging from 131 USD per household in the hot-dry season to 698 USD per household in the wet season in 2023. The technologies introduced in the intervention could be applied to other crops.

**Table 7:** Impact on the revenues of target vegetables by season, in USD per farmer.

Impact	Yardlong beans		Tomatoes		Brassicas		Target vegetables	
	2023	2024	2023	2024	2023	2024	2023	2024
**Hot-dry**								
PSM	52.47*** (13.99)	25.69***(8.80)	1.46(1.25)	0.74(0.74)	9.08(8.53)	−3.52(5.44)	131.17***(34.16)	41.72**(21.07)
IPW	53.12***(13.341)	27.99***(8.18)	–	–	7.53(4.92)	−4.70(5.21)	132.50***(32.06)	36.76*(20.94)
**Wet**								
PSM	302.73***(45.86)	118.60***(26.08)	18.26*(9.83)	10.25(4.25)	−17.04(79.69)	50.16***(18.02)	698.04***(132.20)	437.58***(66.83)
IPW	300.14***(45.82)	130.94***(26.60)	23.88***(9.21)	–	5.08(35.82)	49.92***(16.87)	794.05***(107.73)	462.58***(72.32)
**Regular**								
PSM	153.15***(39.12)	70.21(46.62)	−3.35(18.28)	3.72(2.05)	−2.98(20.93)	10.71(20.27)	259.53***(98.74)	206.01**(93.63)
IPW	152.90***(38.93)	72.80(53.20)	2.35(12.42)	–	2.22(14.84)	12.67(20.79)	352.19***(81.74)	226.77**(103.12)

Notes: *p < 0.1, ** p < 0.05, *** p < 0.01. Coefficients for average treatment effects (ATE) from PSM are presented with standard errors in parentheses. IPW did not converge. The brassica group includes Chinese cabbage, choysum, pak choy, Chinese mustard, headed cabbage, broccoli, cauliflower, radish, and kale. n = 351 farmers per year

## Discussion

This study offers insights into farmers’ adoption of off-season vegetable production after receiving training and some inputs. Almost all farmers in the intervention group attended training sessions covering topics related to each of the three crops of interest from 2021 to 2023, expressing high satisfaction with the modules. Positive and statistically significant effects were observed on the adoption of technologies among the trained farmers. Additionally, the study found that the intervention increased the number of farmers growing yardlong beans in all seasons, as well as tomatoes and brassicas during the wet season.

A qualitative study was conducted in March 2022, halfway to the first endline, to gain a more in-depth understanding of how the technology affected vegetable farming [26]. That study showed that yardlong beans were reportedly grown year-round and fetched good prices, especially during the wet season. Brassicas were also considered important, but farmers viewed these crops as requiring more intensive management, being more susceptible to pests, and producing lower yields. Regarding the technologies discussed in the qualitative study, tomato grafting was a new technology, and only a few farmers adopted it. It was reported that farmers wanted to observe this technology first before trying it themselves. The adoption of grafting may increase over time. Farmers expressed strong concerns about competition from vegetables imported from Vietnam, noting that brassicas and tomatoes were imported in large quantities, were cheaper than locally produced ones, and appeared fresher, making them more appealing to consumers.

Our results showed that the quantity of yardlong beans harvested in all seasons increased in the intervention group, which aligns with evidence from India and Taiwan, where technologies like protected cultivation and drip irrigation have been shown to boost production [14,15]. Off-season production of tomatoes and brassicas was not as successful as yardlong beans. This is likely due to what was reported in the qualitative survey, that brassica crops required more labor and had more issues with pests, and that some farmers were hesitant to adopt tomato grafting before seeing their peers try this method. Regarding other introduced practices, it was reported that the necessary supplies were unavailable locally, and farmers did not want to incur extra costs [26]. This may explain the slightly lower adoption of technologies in the second endline compared to the first. This could be a challenge for sustainability, as evidenced in Nepal, where it was found that having access to materials to use the technologies is necessary to sustain technology adoption for off-season production [10].

Farmers in the sample still face significant constraints in vegetable farming. The three most frequent constraints for wet and hot-dry seasons are the high incidence of insect pests and diseases, low selling prices, and low yields. Flooded fields and too high temperatures are common challenges in wet and hot-dry seasons. However, this analysis shows that training in off-season technologies can increase the use of those technologies and the production of crops in the off-seasons, leading to increased income. There is still more to do to ensure farmers have access to a market for their produce. This is another key to sustaining technology adoption for off-season production, as was also observed in Nepal [10].

This study comes with several strengths. First, with the inclusion of a second endline, this study shows the intervention’s impact is sustained for at least one year after training support ended. Technology adoption was sustained, as was the increase in yardlong bean output. Second, the use of PSM reduces selection bias by creating a more balanced comparison between intervention and control groups by matching similar farmers. Third, the similar results from combining PSM and IPW methods increased confidence in the findings.

However, there were also some limitations to this study. First, if the baseline data were more detailed, then a difference-in-differences estimator could have been applied, which is better at controlling for selection bias or other unobservable characteristics. Second, data on net income were not collected in the surveys. Therefore, while the intervention had positive and significant impacts on vegetable income, there could be substitution effects with other farm activities. Therefore, the results cannot say whether net household income improved. Both have implications for causal inference. The sensitivity analysis does not suggest that the results were driven by unobserved characteristics. Third, the intervention combined training and the supply of inputs, making it difficult to untangle which observed impacts were attributable to training versus inputs. The second endline provides some insight into the farmers’ knowledge gain from the training, as they did not receive additional inputs in the second year. Future research could include more study arms to better understand the impact of each aspect of the intervention. Lastly, the lower adoption rates of brassica and tomato could have been due to other reasons, such as price fluctuations, market access, and supply chain barriers, which were not analyzed in this study.

## Conclusion

Off-season vegetable production technologies are available but not widely adopted in Cambodia and other lower-income countries. Some are simple (e.g., heat-tolerant varieties, plastic mulches), while others are more complex (e.g., vegetable grafting, protected cultivation). This study demonstrated that training farmers in off-season vegetable technologies targeting the hot-dry and wet seasons, and providing them with a starter kit of inputs, increased vegetable production and farm revenue. These benefits persisted for at least one year after the intervention ended. Growing vegetables during the off-season is crucial for creating more stable income for smallholder farmers and ensuring a more consistent market supply of vegetables for consumers.

The model used in this study, with lead farmers training groups of 10–20 neighbors from their village, is a promising example of an intervention that could be scaled up with minimal marginal costs. There is significant potential for applying these technologies in other countries. However, the challenges identified by this study for success include ensuring affordable, available inputs after the starter input kit is used, and a functioning market to which farmers can sell their additional produce. This study indicates that this could be a cost-effective method for improving the year-round supply of vegetables and the livelihoods of smallholder farmers. A cost-effectiveness study would quantify these results for policymakers and provide additional evidence to support future stakeholder investment.

## Supporting information

S1 Table Logit regression results and results of balancing test after matching. (DOCX)

S2 Table Sensitivity of the average treatment effects to hidden bias as based on the bounds test, p-values. (DOCX)

S1 Fig Kernel density distribution showing overlap between intervention and control households, 2023. (TIF)

S2 Fig Kernel density distribution showing overlap between intervention and control households, 2024. (TIF)

S3 Table Sensitivity tests for average treatment effects to alternative matching methods. (DOCX)
