# Supplementary material for: Grow against the flow: The impact of off-season vegetable production in Cambodia
Source: PLoS One. 2026 Sep 16;21(9):e0350969. doi: 10.1371/journal.pone.0350969 (PMC13581017; doi:10.1371/journal.pone.0350969)
Supplement: Supplementary file 1 [file pone.0350969.s001.docx]

**Table S1 Logit regression results and results of balancing test after matching**

a. 2023

| Covariate | Logit regression (n=351) | | | Balancing after matching | | |
| --- | --- | --- | --- | --- | --- | --- |
|  | Coefficient | SE | Sign. | t-value | Sign. | % bias |
| Age | -0.034 | 0.010 | *** | -0.81 | ns | -8.2 |
| Female | -1.159 | 0.234 | *** | 0.23 | ns | 2.4 |
| Province | -0.185 | 0.142 | ns | 0.00 | ns | 0.0 |
| Farmland (ha) | 0.061 | 0.079 | ns | -1.41 | ns | -19.1 |
| Head edu. | -0.036 | 0.040 | ns | 0.30 | ns | 2.9 |
| Pseudo R2 | 0.073 |  |  | 0.006 |  |  |
| Mean abs bias |  |  |  |  |  | 6.5 |

b. 2024

| Covariate | Logit regression (n=351) | | | Balancing after matching | | |
| --- | --- | --- | --- | --- | --- | --- |
|  | Coefficient | SE | Sign. | t-value | Sign. | % bias |
| Age | -0.044 | 0.011 | *** | -0.20 | ns | -2.3 |
| Female | -1.024 | 0.246 | *** | -0.32 | ns | -3.6 |
| Province | -0.098 | 0.142 | ns | -0.90 | ns | -9.9 |
| Farmland (ha) | 0.011 | 0.087 | ns | -0.54 | ns | -6.1 |
| Head edu. | -0.067 | 0.038 | * | 1.66 | * | 17.6 |
| Pseudo R2 | 0.065 |  |  | 0.009 |  |  |
| Mean abs bias |  |  |  |  |  | 7.9 |
