# Supplementary material for: Grow against the flow: The impact of off-season vegetable production in Cambodia
Source: PLoS One. 2026 Sep 16;21(9):e0350969. doi: 10.1371/journal.pone.0350969 (PMC13581017; doi:10.1371/journal.pone.0350969)
Supplement: Supplementary file 2 [file pone.0350969.s002.docx]

**Table S2. Sensitivity of the average treatment effects to hidden bias as based on the bounds test, p-values.**

a. 2023

| Gamma | Technologies adopted | Yardlong beans (kg) in wet season | Brassica (kg) in wet season | Tomatoes (kg) in wet season | Annual vegetable income |
| --- | --- | --- | --- | --- | --- |
| 1.0 | 0.000 | 0.000 | 0.892 | 0.000 | 0.000 |
| 1.2 | 0.000 | 0.000 | 0.733 | 0.001 | 0.002 |
| 1.4 | 0.000 | 0.000 | 0.541 | 0.002 | 0.022 |
| 1.6 | 0.000 | 0.000 | 0.365 | 0.003 | 0.100 |
| 1.8 | 0.000 | 0.000 | 0.229 | 0.005 | 0.256 |
| 2.0 | 0.000 | 0.000 | 0.136 | 0.007 | 0.463 |
| 2.2 | 0.000 | 0.000 | 0.077 | 0.010 | 0.662 |
| 2.4 | 0.000 | 0.000 | 0.042 | 0.013 | 0.812 |
| 2.6 | 0.000 | 0.000 | 0.022 | 0.016 | 0.906 |
| 2.8 | 0.000 | 0.000 | 0.012 | 0.019 | 0.957 |
| 3.0 | 0.000 | 0.000 | 0.006 | 0.023 | 0.981 |

Note: gamma is the log odds of differential assignment due to unobserved covariates. The p-values refer to the upper bound significance levels for overestimation of the treatment effects.

b. 2024

| Gamma | Technologies adopted | Yardlong beans (kg) in wet season | Brassica (kg) in wet season | Tomatoes (kg) in wet season | Annual vegetable income |
| --- | --- | --- | --- | --- | --- |
| 1.0 | 0.000 | 0.000 | 0.000 | 0.002 | 0.007 |
| 1.2 | 0.000 | 0.000 | 0.001 | 0.005 | 0.080 |
| 1.4 | 0.000 | 0.000 | 0.005 | 0.008 | 0.292 |
| 1.6 | 0.000 | 0.001 | 0.015 | 0.013 | 0.578 |
| 1.8 | 0.000 | 0.004 | 0.034 | 0.017 | 0.802 |
| 2.0 | 0.000 | 0.016 | 0.062 | 0.022 | 0.925 |
| 2.2 | 0.000 | 0.044 | 0.101 | 0.028 | 0.976 |
| 2.4 | 0.000 | 0.094 | 0.149 | 0.033 | 0.993 |
| 2.6 | 0.000 | 0.168 | 0.205 | 0.040 | 0.998 |
| 2.8 | 0.000 | 0.264 | 0.265 | 0.045 | 0.999 |
| 3.0 | 0.000 | 0.371 | 0.329 | 0.051 | 0.999 |

Note: gamma is the log odds of differential assignment due to unobserved covariates. The p-values refer to the upper bound significant levels for overestimation of the treatment effects.
