# Supplementary material for: Grow against the flow: The impact of off-season vegetable production in Cambodia
Source: PLoS One. 2026 Sep 16;21(9):e0350969. doi: 10.1371/journal.pone.0350969 (PMC13581017; doi:10.1371/journal.pone.0350969)
Supplement: Supplementary file 3 [file pone.0350969.s005.docx]

**Table S3. Sensitivity tests for average treatment effects to alternative matching methods.**

a. 2023

| Outcome variable | Technologies adopted | Yardlong beans (kg) in wet season | Brassica (kg) in wet season | Tomatoes (kg) in wet season | Annual vegetable income |
| --- | --- | --- | --- | --- | --- |
| IPW | 4.33 | 716.90 | 30.98 | 46.17 | 959.31 |
| PSM (NN, n=1) | 4.32 | 684.30 | -22.73 | 36.47 | 977.70 |
| PSM (NN, n=3) | 4.32 | 711.79 | -2.85 | 51.28 | 993.52 |
| PSM (NN, n=5) | 4.34 | 701.69 | 13.79 | 50.14 | 975.00 |
| PSM (NN, n=1, overlap) | 4.32 | 680.06 | -23.53 | 37.76 | 1001.59 |
| PSM (NN, n=3, overlap) | 4.37 | 714.70 | -4.73 | 53.10 | 1024.77 |
| PSM (NN, n=5, overlap) | 4.38 | 703.59 | 7.79 | 51.92 | 999.65 |
| Coefficient of variation (%) | 0.6 | 2.1 | -10733.2 | 14.8 | 2.2 |

b. 2024

| Outcome variable | Technologies adopted | Yardlong beans (kg) in wet season | Brassica (kg) in wet season | Tomatoes (kg) in wet season | Annual vegetable income |
| --- | --- | --- | --- | --- | --- |
| IPW | 3.29 | 343.38 | 102.42 | - | 1120.12 |
| PSM (NN, n=1) | 3.34 | 310.22 | 107.58 | 19.79 | 805.36 |
| PSM (NN, n=3) | 3.35 | 321.10 | 83.82 | 17.61 | 964.14 |
| PSM (NN, n=5) | 3.28 | 317.86 | 106.73 | 15.81 | 1065.54 |
| PSM (NN, n=1, overlap) | 3.40 | 319.54 | 112.13 | 20.73 | 893.90 |
| PSM (NN, n=3, overlap) | 3.34 | 326.85 | 87.68 | 18.45 | 1044.38 |
| PSM (NN, n=5, overlap) | 3.30 | 324.15 | 109.10 | 16.56 | 1135.40 |
| Coefficient of variation (%) | 1.3 | 3.2 | 11.0 | 10.4 | 12.2 |
